# Global Elimination of HCV—Why Is Poland Still So Far from the Goal?

**DOI:** 10.3390/v15102067

**Published:** 2023-10-09

**Authors:** Olga Tronina, Mariusz Panczyk, Dorota Zarębska-Michaluk, Joanna Gotlib, Piotr Małkowski

**Affiliations:** 1 Department of Transplantation Medicine, Nephrology, and Internal Diseases, Medical University of Warsaw, 02-006 Warsaw, Poland; olga.tronina@wum.edu.pl; 2 Department of Education and Research in Health Sciences, Faculty of Health Sciences, Medical University of Warsaw, 02-091 Warsaw, Poland; joanna.gotlib@wum.edu.pl; 3 Department of Infectious Diseases and Allergology, Jan Kochanowski University, 25-317 Kielce, Poland; dorota1010@tlen.pl; 4 Department of Surgical, and Transplantation Nursing, and Extracorporeal Therapies, Medical University of Warsaw, 02-007 Warsaw, Poland; piotr.malkowski@wum.edu.pl

**Keywords:** chronic hepatitis C, direct-acting antivirals, elimination of hepatitis C virus infection, national action plan for eliminating hepatitis C

## Abstract

Introduction: Eradication of HCV in the global population remains one of the greatest challenges faced by the WHO. An insufficient level of knowledge and the lack of a national screening test strategy are obstacles to HCV eradication. Aim: This work aimed to summarize surveys assessing risk factors and awareness of the respondents regarding the prevention and course of HCV infection. The summary also includes the most important European and global attempts at eliminating HCV. Materials and Methods: A cross-sectional, population-based study was conducted in the Mazowieckie district in Poland using anonymous surveys and conducted on people who willingly reported for a test. Results: In the study cohort of n = 7397 adults, there were 5412 women (73.16%). The analysis of the quota sample (*n* = 1303) reflected the actual proportions in the population of the Mazowieckie Voivodeship. Conclusions: Insufficient knowledge about HCV decreases the probability of higher detection of infections, fast diagnostics, and treatment. According to the WHO model, assuming a 90% detection rate and treatment of 80% of infected by 2030, and taking into account 120–150 thousand infected persons in Poland, the number of detections of HCV should be increased 4–5 times and all diagnosed persons should be offered antiviral treatment.

## 1. Introduction

The eradication of hepatitis C virus (HCV) infection in the world population remains one of the greatest challenges for the World Health Organization (WHO) [1]. This goal is becoming a possibility owing to the introduction of highly effective and safe direct-acting antiviral agents [2,3].

According to the data of the Polish Association for the Study of the Liver and the Polish Group of Experts for HCV (PGE HCV), the estimated number of persons infected chronically with HCV in Poland is approximately 120–150 thousand. Maintaining a number of twelve thousand treated patients per year over the next 10 years, as it was determined for 2017, would fully implement the WHO strategy [4,5].

Unfortunately, consecutive years have brought a systematic decrease in the number of treated patients, and the main issue is not the lack of access to antiviral treatments, but rather the lack of new patients with diagnosed HCV.

This is caused not only by the subclinical form of chronic HCV, which makes it difficult for those infected to identify the problem but also, and most importantly, by the lack of a national strategy for screening tests and an insufficient level of knowledge in the society regarding risk factors, transmission and the possibilities of free diagnosing and treatment of HCV.

With specialized knowledge and the possibility for unlimited testing leading to the detection of numerous new infections and new treatments, there would be a chance to achieve positive effects of such a strategy on multiple levels. The prevention of liver damage through the achievement of a sustained virological response (SVR) would decrease the number of complications of a chronic condition, and the mortality rate resulting from the decompensation of a cirrhotic liver and hepatocellular carcinoma. At the same time, the quality of life of the treated patients would improve. All of these factors would mean that the total economic cost of HCV would decrease [6].

As can be seen, unlimited access to antiviral treatment is not enough. The document entitled ‘National Action Plan for Eliminating Hepatitis C’ published by the PGE HCV as early as 2005 remains without a response.

This article presents a summary of the fulfillment of the Health Policy Programme: ‘Programme of screening studies for the detection of HCV infections’ for 2018–2019 conducted in cooperation with the Medical University of Warsaw and the Mazowieckie Voivodeship Self-Government.

This study aimed to assess the gap between the social knowledge on the complications of chronic HCV, transmission risk factors, diagnostic and therapeutic possibilities, and the perspective of wide-scope educational activities. The increased awareness could lead to mass screening tests, an understanding of the benefits of a national diagnostic and treatment program, and in turn to the elimination of HCV in Poland.

The study also summarises the most important European and global attempts and suggestions for eliminating HCV.

## 2. Materials and Methods

### 2.1. Design

A cross-sectional, population-based study was conducted in the Mazowieckie district, which is the most populated region of Poland.

### 2.2. Setting

The questionnaire was the basic research tool and assessed the risk factors and knowledge levels of the surveyed persons regarding the prevention and course of infection with HCV (Supplementary Materials Table S1).

In 2020, a translation and validation of the Polish version of the validated Brief Hepatitis C Knowledge Scale (BHCKS_PL), was developed by Balfour in 2009 [7].

After completing the questionnaire, a rapid cassette test for anti-HCV antibodies was performed.

The Health Policy Programme of the Mazowieckie Voivodeship Local Government on free screening tests for HCV was fulfilled in the years 2018–2019 in the Mazowieckie Voivodeship, in large and small cities and villages. The test was carried out in hospitals, clinics, city halls, schools and universities, workplaces, cultural centers, military bases, fire stations, and police stations as well as during mass cultural events, concerts, and sports events.

Surveys were conducted on people who willingly reported for a test, male and female adults, regardless of education level. The surveys were anonymous and could be accessed only by the persons participating in the project.

Concerning data from the annual epidemiologic report from 2017 on HCV, created by the European Centre for Disease Prevention and Control (ECDC) and the report of the National Institute of Public Health in Poland, eight areas of potential HCV infection risk were determined. These are medical procedures which include breaking tissue continuity (surgical procedures, blood draws, dental procedures, endoscopic procedures), cosmetic and aesthetic medicine procedures performed with sharp tools, hospitalizations, sharing personal hygienic and cosmetic utensils (e.g., shaving razor), injections and inhalation of drugs, random sexual encounters with no protection, blood transfusions, diagnosed Human Immunodeficiency Virus (HIV) infections.

In Poland, the highest risk of HCV transmission according to the data from 2016 is related to medical procedures (69.8%) [8]. In Western European countries, according to the ECDC report, the highest risk of HCV infection is related to persons who inject drugs (PWID) This is a significantly higher number than in Poland, where drug-related transmission risk is estimated at 5.6% [9].

### 2.3. Sampling and Sample Size

The study utilized two approaches to sample selection. The first approach was a non-probability selection based on the voluntary participation of individuals participating in events organized under the Health Policy Program of the Mazowieckie Voivodship Self-Government. After being informed about the purpose of the study, willing individuals were included in the study sample. As part of the program implementation, a complete set of data was obtained from 7397 participants. From this cohort, quota sampling was made, which is also a non-probability method that relies on the non-random selection of a proportion of units. The quota selection assumed the proportions of the share of sex, age group, education, and place of residence in accordance with the data of the Central Statistical Office for the Mazowieckie Voivodeship (data as of 31 December 2021). Quota sampling enabled the acquisition of a representative sample of respondents (*n* = 1303). For an assumed sample size of 1303, the margin of error was calculated. Assuming a confidence level of 95% and a proportion of 0.5, the estimated error was determined to be 2.8.

### 2.4. Ethical Consideration

The study was conducted according to the guidelines of the Declaration of Helsinki and approved by the Ethics Committee of the Medical University of Warsaw (KB/159/2018), date of approval of 4 November 2018.

### 2.5. Instrument

The study employed a survey methodology. The questionnaire was comprised of three sections: the Polish version of the validated BHCKS_PL, a section on other sociodemographic variables (including gender, age, place of residence, and education), and a 10-point Visual Analogue Scale (VAS) for self-assessment of knowledge about HCV. The survey assessed their own knowledge about HCV on a 1–10 scale.

The questionnaire consisted of two main parts. The first one contains 13 questions aiming to analyze and assess the respondents’ knowledge of HCV, infection transmission, and factors aiding infection. The second part contains 14 questions on the risk factors that occur for the respondents. The last question verified data on previous HCV infection testing.

The survey also took into account basic data such as gender, age, place of residence, and education level. The analysis of the respondents’ answers was conducted in three stages.

The first stage was based on analyses of answers which were not connected to each other. The analysis of the results regarding risk factors was conducted by adding up the answers—positive, negative, and those where the surveyed person did not have any knowledge. In the section on knowledge about HCV, the analysis was conducted by adding up the correct answers—each such answer scored 1 point, and the maximum obtainable point amount was 13. In the second stage, the analysis took into account relations between individual answers. The extension of these analyses (stage three) was obtaining the answers of a specific sub-group of the respondents, i.e., persons who gave a positive answer to the following question: Have you ever in the past conducted a blood test in order to detect HCV? It can be assumed that this group of people has a higher level of knowledge or had some preconditions to undergo the procedure. The results obtained in this sub-group were compared with other results obtained from the analysis in the first stage.

### 2.6. The HCV Antibody Test

After completion of the questionnaire, a rapid cassette test was administered to determine the presence of anti-HCV antibodies. Testing was performed in the whole blood using rapid anti-HCV kits, detecting antibodies generated against the proteins encoded by all HCV genotypes’ most conserved parts of Core, NS3, NS4, and NS5 regions in the HCV genome, which demonstrate sensitivity of 100% and specificity of 100% according to manufacturer (Türklab, Izmir, Turkey). Each individual with a positive serological test result had blood drawn for HCV RNA molecular assays. The HCV RNA levels were obtained by quantitative PCR assays: COBAS TaqMan HCV v2.0 (Roche Molecular Diagnostics, Pleasanton, CA, USA-detection level < 15 IU/mL). Those with a viral load were referred to a specialist outpatient clinic for further diagnostics and antiviral treatment.

### 2.7. Data Collection

The data were collected using two methods in this study: Paper And Pencil Interview (PAPI) and Computer Assisted Personal Interview (CAPI). The tests have been conducted by nurses, who underwent training before the start of the project, both in performing survey studies as well as performing cassette screening tests. The project coordinator was responsible for the training.

The collection of personal data of patients undergoing genetic testing and the archiving of said data was carried out in accordance with the principles of personal data protection.

### 2.8. Statistical Analysis

The data were analyzed using descriptive statistical methods. Qualitative variables are represented by structure measures including number (n) and frequency (%), while quantitative variables as mean (M) and standard deviation (SD).

The impact of selected variables (predictors) on the level of knowledge about HCV was estimated using multivariable regression analysis. All predictors were entered into the regression model simultaneously. For each independent variable, the unstandardized (b) and standardized (β) regression coefficients were determined, with a 95% confidence interval (95% CI) calculated using the bootstrap percentiles method. The parameters of the regression equation were estimated using the least squares method. The assumptions for the linear regression model were satisfied, as indicated by the Ramsey Regression Equation Specification Error Test, White’s test, and the Jarque-Bera test. The presence of autocorrelation was also evaluated by calculating the variance inflation factor (VIF). The adjusted coefficient of determination (R^2^adj.) was calculated. Data were analyzed using IBM SPSS (Version 28) for the descriptive analyses, and Jamovi (Version 2.3.0) to test the regression model. For all analyses, a *p*-value less than 0.05 was deemed to be statistically significant.

## 3. Results

### 3.1. Characteristics of the Study Group

In the study cohort (*n* = 7397), there were 5412 women (73.16%) and 1929 men (26.08%). The remaining ones (*n* = 56; 0.76%) did not provide any information. All individuals completing the survey were adults, ranging in age from 18 to 91 years old. The mean age of the sample was 43.26 (SD = 14.22). Place of residence was indicated by 98.8% of respondents. The largest group (44.94%) of respondents lived in cities with more than 500,000 inhabitants. Cities with up to 50,000 inhabitants were represented by 21.75% of respondents, and villages by 18.75%. As a result of quota sampling, a sample (*n* = 1303) was obtained, for which the distribution of variables such as gender, age group, place of residence, and education reflected the actual proportions in the population of the Mazowieckie Voivodeship (Table 1).

There was no statistically significant difference between the original cohort and the one obtained after quota sampling with regard to the declaration of performing a blood test for detecting hepatitis C in the past (χ^2^ = 1.571, *p* = 0.456).

### 3.2. Risk Factors

**Figure 1:**
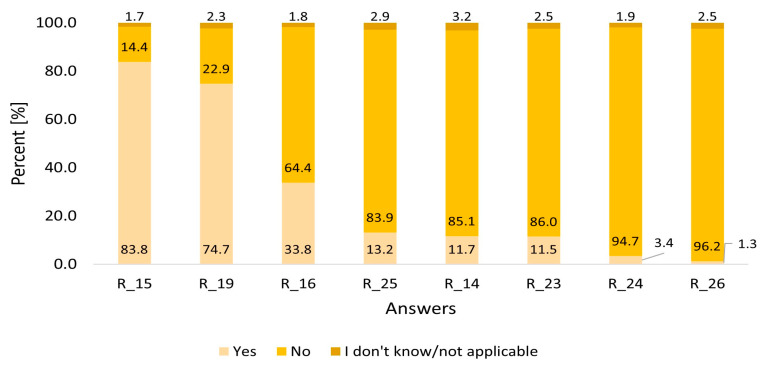
Answers of respondents regarding risk factors (*n* = 7397). R-Question–risk factor assessment. R_15 Have you ever undergone a medical procedure that included the breaking of tissue continuity (surgical procedures, blood draws, dental procedures, endoscopic procedures)? R_19 Have you ever been hospitalized? R_16 Have you ever undergone cosmetic procedures which included sharp implements, and aesthetic medicine procedures such as piercings, tattoos, etc.? R_25 Have you ever had a random sexual encounter with no protection? R_14 Have you ever had a blood or blood product transfusion? R_23 Have you ever shared personal cosmetic-hygenic utensils with other persons (e.g., shaving razors or other sharp cosmetic utensils)? R_24 Have you ever injected or inhaled drugs? R_26 Have you been diagnosed with HIV?

Figure 1 shows a summary of all the survey answers regarding the eight areas of potential HCV transmission risk factors. The surveyed persons had the choice among three answers: Yes, No or I don’t know/Not applicable. The analysis was performed in the available surveyed group (*n* = 7397).

Three risk factors: medical procedures (R15), hospitalization (R19), and cosmetic procedures (R16) were the most frequent answers among the respondents. Various medical procedures were reported by 83,8% of respondents.

One in three persons (*n* = 2173, 29.4%) had at least two, and 1085 (14.7%) had three infection risk factors.

In the analysis of the total number of unmodifiable risk factors of HCV infection (questions R 14,15,17,18,19,20,21,22), two or three factors were reported by more than 60% of respondents (Supplementary Materials Table S2). Similarly to the entire group of respondents, breaking of tissue continuity and hospitalization were the most frequently reported factors occurring in 80.7% and 71.5% of respondents respectively. In the analysis of modifiable risk factors of HCV (questions R 16,23,24,25,26), more than 85% of respondents did not have (54.1%) or had only one (33.2%) risk factor (Supplementary Materials Table S3).

### 3.3. Hepatitis C Knowledge

**Figure 2:**
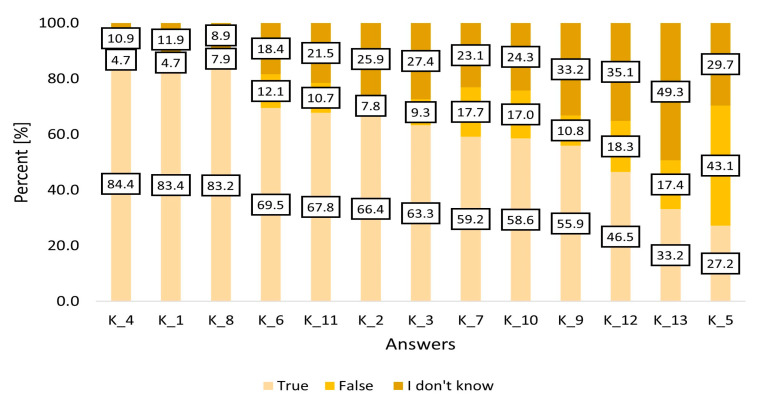
Results of surveys on the awareness about HCV in the cohort after quota sampling (*n* = 1303). K-Question–knowledge assessment. K_4 Alcohol consumption by HCV-infected persons can speed up the process of liver damage. K_1 Chronic HCV can lead to serious complications such as liver cirrhosis and hepatocellular carcinoma. K_8 Using new, never-before-used needles, syringes, and other items decreases the risk of HCV infection. K_6 Persons with HCV can safely share their toothbrushes and shaving razors with other persons. K_11 HCV can be transmitted through coughing, sneezing, handshakes, and hugging. K_2 Due to the asymptomatic course of infection HCV or non-typical symptoms only 1 in 5 persons is aware of infection K_3 Symptoms and results of chronic HCV can affect not only the liver but also other organs such as the heart, kidneys, skin, brain, and pancreas. K_7 HCV can be transmitted during sexual encounters. K_10 HCV can be transmitted through the use of common kitchen utensils (e.g., cups, plates, cutlery, etc.). K_9 Children of mothers with HCV can be infected during labor. K_12 Effective HCV antiviral treatment can lead to a complete eradication of the virus in nearly 100% of patients K_13 Persons with a history of successful HCV antiviral treatment and virus eradication cannot become reinfected with the virus. K_5 HCV vaccination can be used to prevent new infections with the virus.

Figure 2 presents the results of surveys on the awareness of HCV in the cohort after quota sampling (*n* = 1303).

**Figure 3:**
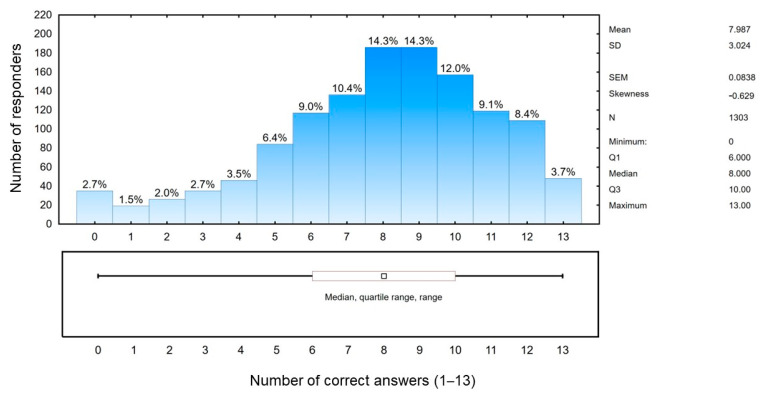
Summarized point results acquired by the respondents reflecting their knowledge of HCV infection.

Based on the answers it can be concluded that more than 80% of the respondents are aware of the consequences of chronic HCV, and also know that alcohol consumption is a risk factor for the disease progression. As little as 27% of the respondents knew the correct answer to the question about HCV vaccination. Knowledge of the risk of repeated infection despite a history of successful antiviral treatment is also low (33.2% of correct answers), and more than half (50.5%) have no knowledge of the possibilities of treating HCV. Figure 3 presents the summarized point result acquired by the respondents reflecting their knowledge of HCV infection.

**Figure 4:**
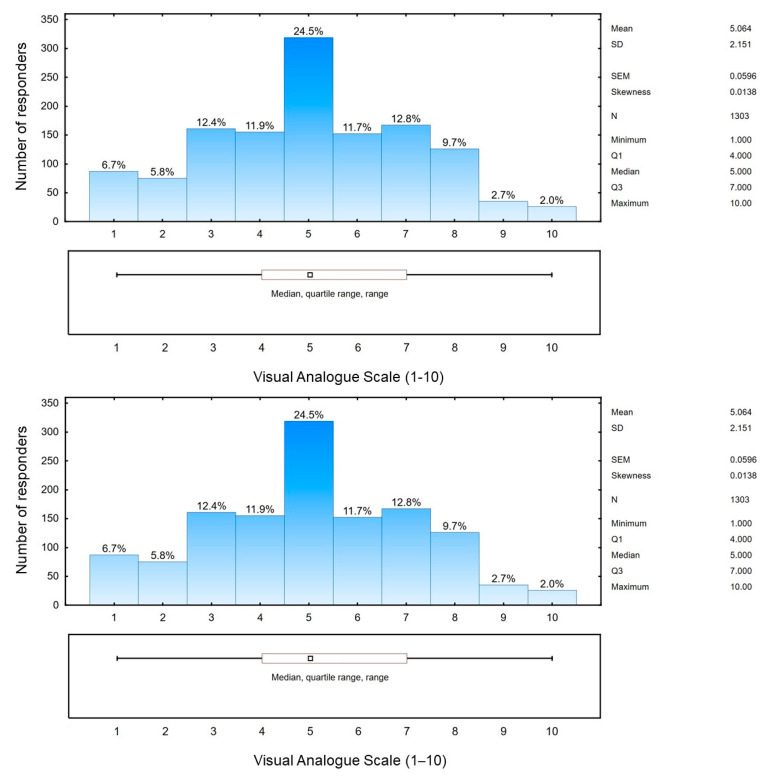
Self-assessment of the respondents’ knowledge of HCV infections (*n* = 1303).

The study participants declared their self-assessment of knowledge in the field of hepatitis C virus infection. The average score obtained by the respondents on the VAS was 5.064 (SD = 2.151) on a scale ranging from 1 to 10. Only 2.0% of respondents indicated a very high level of knowledge, while 6.7% declared a complete lack of knowledge about HCV (Figure 4).

### 3.4. Factors Affecting Hepatitis C Knowledge

The multivariable regression model evaluating the impact of selected factors on the total hepatitis C knowledge was statistically significant (F(12, 1290) = 63.047, *p* < 0.001), and the total explained variance of the dependent variable was R^2^adj. = 36% (Table 2). Among the significant predictors of hepatitis C knowledge, positive predictors included the total number of unmodifiable HCV risk factors (ß = 0.189, *p* < 0.001), previous blood test for hepatitis C (ß = 0.136, *p* < 0.001), higher education (ß = 0.107, *p* < 0.001), and high self-assessment of knowledge (ß = 0.417, *p* < 0.001). Negative predictors included age (ß = −0.111, *p* < 0.001) and lack of knowledge about blood tests for hepatitis C performed in the past (ß = −0.078, *p* = 0.023).

### 3.5. HCV Antibody Test and HCV RNA PCR

During the program of screening tests, a positive result detecting the presence of anti-HCV antibodies was obtained for 20 persons, which constituted 0.27% (*n* = 20/7397) of the tested population. In all persons with positive anti-HCV antibodies a genetic test was performed, identifying the infection in 13 persons (positive HCV RNA PCR, 65% out of 20 seropositive persons).

## 4. Discussion

According to the WHO, HCV kills nearly 300,000 people every year [10]. Persons with chronic infection die usually due to complications—liver cirrhosis and hepatocellular carcinoma (HCC) [10]. Nearly 60 million persons are infected on a global scale with new infections being estimated at 1.5 million per year [10].

There is a chance to alleviate this situation with direct antiviral drugs. Their excellent, near 100%, effectiveness and safety regardless of the degree of liver damage and comorbidities, is a green light in the fight to eliminate HCV, one of the most serious epidemiological threats. However, full eradication of HCV can become a reality once active prevention in the form of vaccinations is added [11].

It has been known for years that HCV is related to the change in the metabolism of glucose. On the one hand, patients with type 2 diabetes can constitute an interesting target group in which more frequent HCV diagnoses can be expected. On the other hand, insulin resistance and diabetes are responsible for the progression of HCV and extra-hepatic manifestations such as nephropathy and Acute cardiovascular incidents. According to data found in literature as many as 33% of patients with chronic HCV are suffering from type 2 diabetes [12,13].

The WHO in a document entitled ‘Global health sector strategy on viral hepatitis 2016-2021. Towards ending viral hepatitis” set a goal of eliminating hepatitis B virus and HCV infections by 2030, diagnosing 90% of the infected persons, and introducing antiviral treatment in 80% of those infected [1]. In 2015, treatment was received by less than 1% of those infected with HCV. According to a different report in 2019, there were 15.2 million people aware of being infected, and 9.4 million of the infected received antiviral treatment in 2015–2019 [14].

Hellard et al., in the publication ‘The Elimination of Hepatitis C as a Public Health Threat’, stress the importance of knowledge about methods of transmission and detection of active HCV infections [15]. This is of fundamental importance for a multifaceted elimination intervention. The awareness of risk factors and the awareness of potential contact with a deadly virus and of the availability of testing, as well as fast and safe treatment can be a motivator for performing a test and reporting for treatment. It is difficult to convince an asymptomatic person, who has never heard of the consequence of chronic HCV, to test themselves. Meanwhile, in the answers provided by the surveyed individuals in our study, doubt is visible in the answers regarding the transmission methods of HCV. There were even fewer correct answers obtained for the questions on treatment and vaccinations. Focused educational and pro-health campaigns could encourage those unaware of the HCV issue to do a screening test. In Poland, through the engagement of the hepatology environment, many diagnostic laboratories offer a free serologic test to identify HCV infections. In this way, the combination of education and engagement becomes a valid tool in an area where systemic solutions have failed.

The analysis of the respondents’ answers shows that >80% of the population is subjected to the most commonly reported risk factor of HCV infection in Poland. This is an argument for the need for mass screening tests of the population.

One of the most dynamic and reference-worthy examples of an effective HCV elimination program, a representative of a low-middle country and, at the same time, a country with one of the highest prevalence of HCV in the world is Egypt [16]. Mass testing of people aged 18–59, a beneficial process of serological and HCV-RNA PCR tests as well as antiviral drugs allowed 79% of planned tests to be performed in under a year. As many as 92% of diagnosed patients began treatment with a sustained virological response of 98%. The total cost of the program reached USD 207 million, but after calculating all of the medical costs and those related to disability and premature death it became apparent that population screening would be very cost-saving.

Similar conclusions were drawn by researchers in Japan. Based on a model reflecting the progression of liver fibrosis and the assessment of the profitability of therapeutic strategies at different stages of fibrosis, and based on comparable analysis from the United States, Suenaga formed the conclusion that the current Japanese recommendations should allow to administer DAA treatment to all those infected with genotype 1 (GT1) regardless of the level of fibrosis. This solution would be the most cost-effective [17].

Therapeutic programs in Polish health care allow the treatment of nearly all infected patients. Despite this, the system of HCV eradication is based on a random identification of persons infected with HCV. What is worse, this most often happens in the case of a significant disease progression, in its advanced stages, when the patient reports to the health care system with severe health problems and complications.

This usually requires the introduction of radical treatment methods, such as liver transplantation followed by many years of immunosuppressive treatment and medical care, or is limited to hospice care in which case a fatal outcome in a short time span is of high probability. This, as a consequence, generates many socio-economic issues, such as the necessity of permanent bearing of patient treatment costs—both in hospital and post-discharge, as well as the cost of treatment of complications. Additionally, the socio-economic costs include the decrease or lack of economic productivity of patients and their families, which is the result of limited professional activity, presenteeism, or the collection of sickness benefits.

As a consequence, the current scenario does not provide a solution that would definitely limit the scale of infection and the range of activities related to the treatment of the outcomes and complications [18,19,20].

It is clear that a radical increase in infection detectability requires the implementation of a strategy that would enable the obtaining of desired detection rates combined with an optimization of its functioning costs.

Flisiak, together with experts from eight Central European countries, considers the insufficient number of diagnosed and treated patients annually, as the largest obstacle to eradicating HCV in this region. This results from the lack of a national screening program, and the lack of political will to implement one [21]. Additional pain points are staffing shortages and the suboptimal organization of the healthcare service. The insufficient number of diagnosed and treated patients is the result of the lack of awareness and knowledge about the disease. The low estimation of knowledge about HCV among the surveyed persons in our study confirms this thesis.

The multivariate analysis shows that this issue is less common among persons with higher education. Most often, the surveyed persons from this group had performed a screening test for HCV in the past; they also assessed their own level of knowledge about HCV as good. All of these countries negate issues related to financing treatment. There also are no queues of patients waiting for treatment. Experts point to the necessity of creating a fast diagnostic and therapeutic path [22]. A similar problem was described by Feld who summarised the meeting of the Special Interest Group convened by The American Association for the Study of Liver Diseases (AASLD) in 2020 [23]. The two-tier screening process which included a waiting period for the results of the molecular test, caused a part of the serologically positive patients to be lost before completing the process which would qualify them for treatment.

The Italian model places stress on the necessity to simplify the HCV elimination scheme through the reduction of follow-up appointments, the introduction of telemonitoring, as well as fast and non-invasive diagnostic processes. Such steps can help reduce the number of patients lost from observation [24]. The authors also state that success can be reached through the engagement of multidisciplinary teams, family doctors, pharmacists, nurses, and social workers

In Poland, the largest screening test projects are the initiative of the Polish Association of Epidemiologists and Infectiologists (PTEiLChZ), the Polish Association for Study of Liver (PASL), and local authorities in cooperation with hepatology experts. Piekarska et al. summarized one of the largest of such projects. In the years 2018–2019, the study included 723,654 persons, and testing was performed in whole blood using rapid anti-HCV kits [25]. The total number of positive results was obtained for 3548 persons, which means a prevalence rate of 0.5%. The highest number of positive test results (1.2%) was obtained from patients in diagnostic labs, who reported there during the HCV awareness campaign. Other places of significant seroprevalence (0.6–0.7%) were screening points, hospitals, tests performed during music events, as well as in private medical centers [25].

According to the results of our survey, more than 80% of the studied persons underwent a medical procedure—the most common HCV infection risk factor in Poland, which would confirm the need for a wide range of population screenings.

This analysis selects the potential area to conduct at least one screening test for every person with a risk factor. A question can be formed whether it is economically feasible, justified, and rational to perform HCV screening tests for all citizens. This would mean taking into account high costs as well as logistic, ethical, and legal difficulties

It therefore seems that selected groups of individuals or places need to be focused, which show the highest probability and risk of HCV infections. However, such actions may not yield the desired effect. In France, a strategy of screening tests only in groups of high risks was implemented [26]. As a result, 40% of those infected with HCV remained undiagnosed. In accordance with recommendations from experts from the French Health Ministry, a wide range of screening processes and antiviral treatment for all persons between the ages of 15 and 75 regardless of the degree of liver fibrosis seems the most cost-effective strategy.

On the other hand, an analysis of German screening tests shows that searching for new infections in risk groups makes it possible to identify 83% of all infected [27]. In Germany, the number of PWID had increased from 26.3% in 2014 to 43.1% in 2018 which constitutes 80% of newly identified HCV infections. This means that HCV elimination attempts in this country should focus on intensified testing of high-risk populations: PWID, inmates, and immigrants from regions with high HCV prevalence. Despite the fact that medical procedures seem to be the main source of HCV infections in Poland, the issue of HCV infections among PWIDs cannot be ignored. According to one of the reports, positive anti-HCV antibodies were identified in 59.5% of PWID [28].

**Figure 5:**
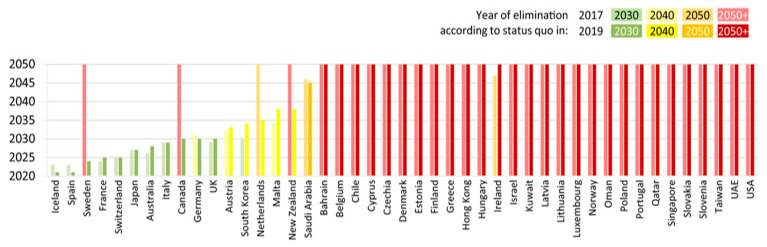
The progress towards the WHO’s 2030 HCV elimination targets in high-income countries from the Polaris observatory [7].

It needs to be stressed that both Germany and France are countries that have the chance to eradicate HCV by the year 2030 (Figure 5). France is basing their actions on three principles: the increase of screening and medical care availability, as well as infection and reinfection prevention.

The issue of reinfection among inmates has been pointed out by Dore who describes an effective path to HCV elimination in Australia [29]. The study suggests the strengthening of primary prevention and the need for more harm reduction, paying attention to expanded treatments with opioid agonists and prison programs regarding needles and syringes.

The last element, due to the lack of possibility for effective vaccinations, is of significant prevalence importance among high-risk populations—those who inject drugs and HIV-positive persons.

Authors of the report on the National Health Program for the years 2016-2020 in Poland while assessing the issues of diagnosing chronic HCV, in the chapter on HCV, have stressed that the current efforts to improve diagnostics were focusing on screening tests for the easily available general population. This seems to be confirmed by a relatively low percentage of seroprevalence (0.27%) in our testing efforts. The increasing percentage of undiagnosed infections can be therefore related to persons from marginalized populations, including PWID [30]. The underestimation of infections as an effect of testing occurring once every 12 months, which is not in accordance with the standards of the European Centre for Infectious Disease Control and Prevention and the European Monitoring Centre for Drugs and Drug Addiction, as well as restrictions in antiviral treatment availability for this population, are additional factors making the elimination of HCV infections in Poland more difficult [31].

Based on the analysis of available literature and the results of original empirical studies, we suggest that the future forming and implementation of strategies for HCV infection eradication in Poland should be based on the guidelines and solutions presented synthetically in Table 3.

The proposed eradication scenario, built according to the recommendations of the WHO has the goal of eliminating the issue of HCV infections in a 10-year perspective. The actions presented in the table above should become the guidelines of a national program of screening tests. It would, as a consequence, have an active structure, presenting a set of necessary activities, which—when undertaken—would provide a chance to halt and eliminate HCV infections. In order to achieve the target goals in Poland approximately 12,000 persons should undergo treatment per year. This means that the current diagnosis rate of 4000 persons per year would have to be increased 3–4 times, to a minimum of 12,000 diagnoses per year. This, in turn, entails the necessity of performing approximately 3 million screening tests per year.

### 4.1. Conclusions

The lack of a national strategy for combating HCV gives us no chance to effectively detect new infections, treat them, increase the risk of transmission, and as a consequence prevent the elimination of HCV in Poland in accordance with the proposed strategy from the WHO. In relation to the potential risk factor of HCV infection through medical procedures, which—according to the analysis—pertains to the majority of the Polish population, as well as limited knowledge of vaccinations, reinfections, and treatment of HCV it needs to be concluded that educational campaigns can be the first important step in a strategy for the elimination of HCV.

Educational campaigns aiming at increasing awareness of HCV, its complications, and effective treatment can increase the number of people who report for screening tests in diagnostic laboratories. These facilities have the highest rate of HCV detection in Poland, according to current data. However, without taking steps toward the implementation of a national screening test program, Poland does not have a chance of eradicating HCV by 2030. Unfortunately, a similar problem is being faced by 80% of high-income countries and the chances are even slimmer in mid- and low-income countries.

The long road towards HCV eradication is a complex issue and requires a series of actions from a multidisciplinary team.

### 4.2. Limitations

The biggest limitation of this study is the lack of connected dependencies between the confirmed infection with HCV in the 13 surveyed patients and their knowledge of risk factors and self-assessment.

This was due to the anonymous form of the survey, and also with consecutive organizational disturbances.

Another limitation is the fact that the study was conducted only in the Mazowieckie Voivodeship which, despite meeting the quota sampling, may not reflect the entire population of Poland. Unfortunately, as was mentioned previously, the lack of a national program and related funding for screening tests allows only for partial projects organized by local governments. This can distort the global assessment of the HCV issue in Poland, despite attempts at quota sampling.

## Figures and Tables

**Table 1:** Baseline characteristics.

Variable	Original Cohort (*n* = 7397)		Cohort after Quota Sampling (*n* = 1303)	
	*n*	%	*n*	%
Gender				
female	5412	73.16	661	50.73
male	1929	26.08	642	49.27
data missing	56	0.76	-	-
Age group				
18–19	191	2.58	57	4.37
20–24	654	8.84	100	7.67
25–34	1227	16.59	280	21.49
35–44	1702	23.01	354	27.17
45–54	1526	20.63	275	21.11
55–64	1273	17.21	237	18.19
data missing	824	11.14	-	-
Place of residence				
village	1387	18.75	461	35.38
cities with up to 50,000 inhabitants	1609	21.75	232	17.81
cities with 51,000–200,000 inhabitants	988	13.36	145	11.13
cities above 200,000 inhabitants	3324	44.94	465	35.68
data missing	89	1.20	-	-
Education				
primary/vocational	701	9.48	154	11.82
high school graduate	2272	30.72	401	30.78
university level	4370	59.08	748	57.41
data missing	54	0.73	-	-
Have you ever had a blood test to detect HCV?				
yes	1568	21.20	281	21.57
no	5273	71.29	912	69.99
I don’t know	556	7.52	110	8.44

**Table 2:** Multivariable regression model explaining the influence of selected predictors on the knowledge of HCV infections (*n* = 1303).

	Level	b	ß	95% CI		*t*	*p*-Value
				Lower Limit	Upper Limit		
Intercept		4.925				15.247	<0.001
Total number NMf		0.512	0.189	0.141	0.237	7.720	<0.001
Total number Mf		−0.038	−0.010	−0.055	0.035	−0.424	0.672
Earlier blood tests	no (ref.)						
	yes	0.500	0.136	0.063	0.209	3.667	<0.001
	I don’t know	−0.371	−0.078	−0.147	−0.009	−2.227	0.026
Gender	Female (ref.)						
	male	−0.129	−0.043	−0.087	0.002	−1.895	0.058
Age		−0.027	−0.111	−0.156	−0.067	−4.917	<0.001
Place of residence	village (ref.)						
	cities with up to 50,000 inhabitants	0.386	0.090	0.028	0.153	2.844	0.005
	cities with 51,000–200,000 inhabitants	−0.076	−0.016	−0.083	0.051	−0.468	0.640
	cities above 200,000 inhabitants	0.071	0.020	−0.042	0.082	0.627	0.531
Education	P/V (ref.)						
	HSG	−0.056	−0.012	−0.055	0.032	−0.518	0.604
	UL	0.464	0.107	0.060	0.154	4.488	<0.001
Self-assessment of knowledge		0.586	0.417	0.370	0.464	17.387	<0.001

b—unstandardized regression coefficient, ß—standardized regression coefficient, CI—confidence interval, ref.—reference level, NMf—non-modifiable factors, Mf—modifiable factors, P/V—primary/vocational, HSG—high school graduate, UL—university level.

**Table 3:** Areas and range of proposed actions in the eradication scenario [4,15,16,21,23,25,26,27,32,33].

Area	Type and Form of Action	Scope of Action/Executors
The organization of the system	Forming and approval of the implementation of a national screening test program	Ministry of Health and the required institutions of the health protection system
Determination of the time period	2023–2033	
Preparation of organization-financial solutions—negotiations of diagnostic tests and antiviral drug processes	Ministry of Health and the National Health Fund (NFZ)	
Strategy implementation	National Health Fund	
Processes	Educational activities and public health campaign	Cooperation with primary and high schools, higher education units, and Universities of Senior CitizensInformation and promotional campaigns in social media, information brochures, educational campaigns through local governments and school boards, informational booths at cultural and sporting events, as well as social media posts.
Location of screening tests	Grassroots initiatives for screening tests.Hospitals, state and private centers, medical procedure units, medical labs and testing points, occupational medicine physicians—every patient and every visit (general population testing and risk groups—people with diabetes, inmates, PWID, men who have sex with other men, the homeless)	
Infection diagnostics	Obligatory HCV cassette test—a fast diagnostic path for persons with a positive serological test, fast genetic testing	
Treatment qualification	Simplified—reduction of qualifying visits and visits during treatment, telemonitoring, no need for genotyping or fibrosis assessment on initial qualificationIncreased staffing	
Treatment of infections	Introduction of a therapeutic program—unlimited access to modern therapies (Glecaprevir/Pibentasvir, Sofosbuvir/Velpatasvir or Sofosbuvir/Velpatasvir/Voxilaprevir)—duration time—maximum 12 weeks—patient-friendly system (reduction of professional absence related to treatment)	
Treatment of complications	In accordance with current procedures	
Indicators	Managing the economic effectiveness of the system	NFZ—monitoring and flexibility (especially regarding the number of selected infections—maintaining the required level of 10–12 thousand infections per year)
Determining Key Performance Indicators (KPI)	The target indicator for eliminating infections in the population—is 90% The target indicator of infected treated using a modern antiviral treatment—80–90%	
Indicator monitoring	Current monitoring of infection indicators decrease	
Expected effects	Corrections of the implemented actions	In the case of not obtaining the minimal number of original (new) patients—expanding the screening tests—the initial target is 3 million screening tests per year
Infected population	Annual decrease of approx. 12,000 persons in the initial group of approx. 120,000 infected	
Treated patients	Minimum 12,000 new cases per year	
Prevention	Expanding the group and number of persons included in screening tests (possible thanks to new funds in the system as an expected result of the lowering of the costs of financing the current system)	
Elimination of infection	Infection elimination level in the population—90–100%Percentage of those infected with HCV undergoing treatment—80–90%	

## Data Availability

The authors confirm that the data supporting the findings of this study are available within the article and its Supplementary Materials.

## Supplementary Materials

The following supporting information can be downloaded at: https://www.mdpi.com/article/10.3390/v15102067/s1, Table S1: The questionnaire; Table S2: The total number of unmodifiable risk factors; Table S3: The total number of modifiable risk factors.

## References

[1] World Health Organization (2016). Global Health Sector Strategy on Viral Hepatitis 2016–2021. Towards Ending Viral Hepatitis. https://apps.who.int/iris/handle/10665/246177.

[2] Pawlotsky J.M., Negro F., Aghemo A., Berenguer M., Dalgard O., Dusheiko G., Marra F., Puoti M., Wedemeyer H. (2020). EASL recommendations on treatment of hepatitis C: Final update of the series. J. Hepatol..

[3] Hepatitis C. (2018). AASLD-IDSA HCV Guidance Panel. Hepatitis C Guidance 2018 Update: AASLD-IDSA Recommendations for Testing, Managing, and Treating Hepatitis C Virus Infection. Clin. Infect Dis..

[4] Flisiak R., Zarębska-Michaluk D. (2019). Perspectives of hepatitis C virus (HCV) elimination in Poland. Clin. Exp. Hepatol..

[5] Razavi H., Gonzalez Y.S., Yuen C., Cornberg M. (2020). Global timing of hepatitis C virus elimination in high-income countries. Liver Int..

[6] Nyberg A.H., Sadikova E., Cheetham C., Chiang K.M., Shi J.X., Caparosa S., Younossi Z.M., Nyberg L.M. (2020). Increased cancer rates in patients with chronic hepatitis C. Liver Int..

[7] Tronina O., Gotlib J., Małkowski P., Jaworski M., Panczyk M. (2020). Translation and validation study of the Polish version of the Brief Hepatitis C Knowledge Scale. PLoS ONE.

[8] Zakrzewska K., Stępień M., Szmulik K., Rosińska M. (2018). Hepatitis C in Poland in 2016. Przegl. Epidemiol..

[9] Ndumbi P., Freidl G.S., Williams C.J., Mardh O., Varela C., Avellón A., European Centre for Disease Prevention and Control (2022). Hepatitis C. Annual Epidemiological Report for 2020.

[10] World Health Organization (2013). Hepatitis C. https://www.who.int/news-room/fact-sheets/detail/hepatitis-c.

[11] Cox A.L. (2020). Challenges and Promise of a Hepatitis C Virus Vaccine. Cold Spring Harb. Perspect. Med..

[12] Gastaldi G., Goossens N., Clément S., Negro F. (2017). Current level of evidence on causal association between hepatitis C virus and type 2 diabetes: A review. J. Adv. Res..

[13] Hammerstad S.S., Grock S.F., Lee H.J., Hasham A., Sundaram N., Tomer Y. (2015). Diabetes and Hepatitis C: A Two-Way Association. Front. Endocrinol..

[14] World Health Organization 2021 Global Progress Report on HIV, Viral Hepatitis and Sexually. https://www.who.int/publications/i/item/9789240027077.

[15] Hellard M., Schroeder S.E., Pedrana A., Doyle J., Aitken C. (2020). The Elimination of Hepatitis C as a Public Health Threat. Cold Spring Harb. Perspect. Med..

[16] Waked I., Esmat G., Elsharkawy A., El-Serafy M., Abdel-Razek W., Ghalab R., Elshishiney G., Salah A., Megid S.A., Kabil K. (2020). Screening and Treatment Program to Eliminate Hepatitis C in Egypt. N. Engl. J. Med..

[17] Suenaga R., Suka M., Hirao T., Hidaka I., Sakaida I., Ishida H. (2021). Cost-effectiveness of a “treat-all” strategy using Direct-Acting Antivirals (DAAs) for Japanese patients with chronic hepatitis C genotype 1 at different fibrosis stages. PLoS ONE.

[18] Wong W.W., Haines A., Bremner K.E., Yao Z., Calzavara A., Mitsakakis N., Kwong J.C., Sander B., Thein H.-H., Krahn M.D. (2021). Health care costs associated with chronic hepatitis C virus infection in Ontario, Canada: A retrospective cohort study. CMAJ Open.

[19] Razavi H., ElKhoury A.C., Elbasha E., Estes C., Pasini K., Poynard T., Kumar R. (2013). Chronic hepatitis C virus (HCV) disease burden and cost in the United States. Hepatology.

[20] Younossi Z.M., Stepanova M., Henry L., Younossi I., Weinstein A., Nader F., Hunt S. (2016). Association of work productivity with clinical and patient-reported factors in patients infected with hepatitis C virus. J. Viral Hepat..

[21] Flisiak R. (2020). Eliminacja zakażeń wirusem zapalenia wątroby typu C w Europie Środkowej. Hepatologia.

[22] Flisiak R., Frankova S., Grgurevic I., Hunyady B., Jarcuska P., Kupčinskas L., Makara M., Simonova M., Sperl J., Tolmane I. (2020). How close are we to hepatitis C virus elimination in Central Europe?. Clin. Exp. Hepatol..

[23] Feld J.J., Ward J.W. (2021). Key Elements on the Pathway to HCV Elimination: Lessons Learned from the AASLD HCV Special Interest Group 2020. Hepatol. Commun..

[24] Di Marco L., La Mantia C., Di Marco V. (2022). Hepatitis C: Standard of Treatment and What to Do for Global Elimination. Viruses.

[25] Piekarska A., Tomasiewicz K., Halota W., Jaroszewicz J., Krygier R., Małkowski P., Pawłowska M., Simon K., Tronina O., Zarębska-Michaluk D. (2020). Searching for the optimal population for hepatitis C virus screening in Poland. Clin. Exp. Hepatol..

[26] Pol S., Lair-Mehiri L., Vallet-Pichard A. (2021). Is elimination of HCV realistic by 2030: France. Liver Int..

[27] Sarrazin C., Boesecke C., Golsabahi-Broclawski S., Moog G., Negro F., Silaidos C., Patel P., Lohmann K., Spinner C.D., Walcher S. (2021). Hepatitis C virus: Current steps toward elimination in Germany and barriers to reaching the 2030 goal. Health Sci. Rep..

[28] Rosińska M., Zieliński A. (2004). Raport z Programu Badawczego “Oszacowanie Występowania Chorób Zakaźnych (Wirusowe Zapalenie Wątroby Typu C i B, HIV) Wśród Narkomanów Przyjmujących Środki Odurzające w Iniekcji w Miastach o Różnym Stopniu Realizacji Programów Redukcji Szkód”. Zakład Epidemiologii Państwowego Zakładu Higieny Warszawa. https://www.cinn.gov.pl/.

[29] Dore G.J. (2021). Editorial: Elimination of hepatitis C in Australia by 2030: A decade and counting. Aust. Prescr..

[30] Wojtyniak B., Goryński P. (2020). Sytuacja Zdrowotna Ludności Polski i jej uwarunkowania 2020. Narodowy Instytut Zdrowia Publicznego.

[31] (2019). European Centre for Disease Prevention and Control Hepatitis C. Annual Epidemiological Report for 2017.

[32] Tomasiewicz K., Flisiak R., Jaroszewicz J., Małkowski P., Pawłowska M., Piekarska A., Simon K., Zarębska-Michaluk D. (2023). Recommendations of the Polish Group of Experts for HCV for the treatment of hepatitis C in 2023. Clin. Exp. Hepatol..

[33] Cox A.L., El-Sayed M.H., Kao J.-H., Lazarus J.V., Lemoine M., Lok A.S., Zoulim F. (2020). Progress towards elimination goals for viral hepatitis. Nat. Rev. Gastroenterol. Hepatol..

