# Supplementary material for: Global Elimination of HCV—Why Is Poland Still So Far from the Goal?
Source: Viruses. 2023 Oct 9;15(10):2067. doi: 10.3390/v15102067 (PMC10612042; doi:10.3390/v15102067)
Supplement: Supplementary file 1 [file viruses-15-02067-s001.zip › viruses-2639417-supplementary.pdf]

Table S1. The questionnaire

	Statement – knowledge assessment	Yes	No	I don't know
**K1**	Chronic HCV can lead to serious complications such as liver cirrhosis and hepatocellular carcinoma.	☐	☐	☐
**K2**	Due to the asymptomatic course of infection HCV or non-typical symptoms only 1 in 5 persons is aware of infection	☐	☐	☐
**K3**	Symptoms and results of chronic HCV can affect not only the liver but also other organs such as: heart, kidneys, skin, brain and pancreas.	☐	☐	☐
**K4**	Alcohol consumption by HCV infected persons can speed up the process of liver damage.	☐	☐	☐
**K5**	HCV vaccination can be used to prevent new infections with the virus.	☐	☐	☐
**K6**	Persons with HCV can safely share their toothbrushes and shaving razors with other persons.	☐	☐	☐
**K7**	HCV can be transmitted during sexual encounters.	☐	☐	☐
**K8**	Using new, never before used needles, syringes and other items decreases the risk of HCV infection.	☐	☐	☐
**K9**	Children of mothers with HCV can be infected during labour.	☐	☐	☐
**K10**	HCV can be transmitted through the use of common kitchen utensils (e.g. cups, plates, cutlery, etc.).	☐	☐	☐
**K11**	HCV can be transmitted through coughing, sneezing, handshakes and hugging.	☐	☐	☐
**K12**	Effective HCV antiviral treatment can lead to a complete eradication of the virus in nearly 100% of patients.	☐	☐	☐
**K13**	Persons with a history of successful HCV antiviral treatment and virus eradication cannot become reinfected with the virus.	☐	☐	☐

K - knowledge

	Question – risk factor assessment	Yes	No	I don't know/Not applicable
**R14**	Have you ever had a blood or blood product transfusion?	☐	☐	☐
**R15**	Have you ever undergone a medical procedure which included the breaking of tissue continuity (surgical procedures, blood drawing, dental procedures, endoscopic procedures)?	☐	☐	☐
**R16**	Have you ever undergone consmetic procedures which included wharp implements, aesthetic medicine procedures such as piercings, tattoos, etc.?	☐	☐	☐
**R17**	Are you undergoing long-term dialysis?	☐	☐	☐

**R18**	Are you suffering from haemophilia?	☐	☐	☐
**R19**	Have you ever been hospitalized?	☐	☐	☐
**R20**	Is your work related to occupational exposure to blood product pathogens?	☐	☐	☐
**R21**	Have you ever been exposed to blood product pathogens at work?	☐	☐	☐
**R22**	Have you been diagnosed with HBV and/or HCV during pregnancy?	☐	☐	☐
**R23**	Have you ever shared personal cosmetic-hygenic utensils with other persons (e.g. shaving razors or other sharp cosmetic utensils)?	☐	☐	☐
**R24**	Have you ever injected or inhaled drugs?	☐	☐	☐
**R25**	Have you ever had a random sexual encounter with no protection?	☐	☐	☐
**R26**	Have you been diagnosed with HIV?	☐	☐	☐
**R27**	Have you ever performed a blood test for HCV?	☐	☐	☐

R – risk factor

Personal Information:

1. **Gender**

   ☐ Female ☐ Male

2. **Age:** ☐☐

3. **Place of residence**

   ☐ Village

   ☐ City under 50,000 inhabitants ☐ City between 51,000 and 200,000 inhabitants

   ☐ City between 201,000 and 500,000 inhabitants

   ☐ City above 500,000 inhabitants

4. **Education**

   ☐ Primary/Vocational ☐ High School graduate

   ☐ Higher education

   ☐ Other

5. **How do you assess your knowledge on HCV:**

1 – no knowledge

|__1__|__2__|__3__|__4__|__5__|__6__|__7__|__8__|__9__|__10__|

10-very high level of knowledge

Table S2. The total number of unmodifiable risk factors

	The total number of unmodifiable risk factors	
	N	%
0	130	9.98
1	218	16.73
2	532	40.83
3	268	20.57
4	135	10.36
5	20	1.53

Table S3 The total number of modifiable risk factors

	The total number of modifiable risk factors	
	N	%
0	706	54.18
1	433	33.23
2	130	9.98
3	29	2.23
4	5	0.38
